# From small batteries to big claims

**DOI:** 10.1038/s41565-025-01906-3

**Published:** 2025-04-11

**Authors:** Rares-George Scurtu, Alessandro Innocenti, Vanessa Scheck, Mario Maunz, Thomas Waldmann, Markus Hölzle, Alice Hoffmann, Peter Axmann, Margret Wohlfahrt-Mehrens

**Affiliations:** 1 Zentrum für Sonnenenergie- und Wasserstoff-Forschung Baden-Württemberg, Ulm, Germany; 2 University of Ulm, Ulm, Germany

**Keywords:** Batteries, Engineering, Electrochemistry, Energy science and technology, Energy storage

## Abstract

A frequently undervalued aspect of lithium-ion battery performance reporting is the specification of the format and area of the tested cells. However, these parameters provide crucial insights into the quality of the electrodes used for cell assembly and the reliability of the data obtained from the investigated systems. Here we focus on the aspects of process standardization and industry collaboration necessary for translating nanoscale electrochemical processes to Ah-scale cells. We examine the role of cell area and format in promoting comparability and standardization in battery research studies with technology readiness levels of 4 or higher. In addition, we discuss the limitations, challenges and expectations associated with measuring and evaluating battery performance exclusively in small cell formats.

## Abstract (web summary)

In battery research, the areas of the electrodes and cell dimensions affect the energy storage performance. Here the authors discuss the factors that influence the reliability of electrochemical measurements and battery performance in lithium-ion cells with different electrode areas.

## Main

Standardization in battery performance reporting is crucial for ensuring consistency and comparability of data across scientific studies on this topic^1^. Many studies often use experimental conditions that differ substantially from practical applications, such as low active material loading, large electrolyte volumes and low charging current rates. Standardizing data reporting practices would clarify the conditions under which batteries are tested and help to identify the most promising and impactful research. Some scholarly journals have made efforts in this direction by requiring authors to complete a detailed checklist concerning, for example, electrode properties and cycling conditions^2–4^. Recently, several interlaboratory collaborations have been conducted to assess the reproducibility of experiments on electrochemical devices made from the same materials but carried out in different settings, often revealing wide variations in the reported results^5–7^. Within the Battery 2030+ project, a large-scale and long-term European research initiative, scientists and researchers developed an online, collaborative, open-access platform called the Battery Knowledge Base (https://battery.knowledge-graph.eu/wiki/Main_Page), which documents all steps of battery manufacturing alongside related process parameters and measurable quantities. These initiatives aim to harmonize materials research studies, which very often use coin cells or similar formats (with a technology readiness level (TRL) ≤3)^8^. However, to the best of our knowledge, the scientific community has not yet explicitly addressed the standardization of battery performance reporting for research studies with a TRL ≥4.

In this Analysis, building on the extensive experience we have gained in upscaling the fundamental electrochemical energy storage processes of the battery, which occur at the nanoscale^9^, to the Ah scale^10–16^, we aim to highlight the importance of detailing the geometric area and format of lithium-ion cells. These aspects are often overlooked in battery research operating at TRL ≤3, while they are of particular importance in studies with a TRL ≥4. We also propose a framework for reporting these parameters and present results from an experimental campaign involving more than 30 21700-type cylindrical cells, all using the same materials and testing procedures. In doing so, we aim to emphasize the importance of considering aspects such as the reliability of results in terms of capacity, applied potential, resistance and cycling stability as well as the suitability of large-area wound cells, that is, cells where the electrodes and separators are wound around a winding mandrel or centre pin, as a better standard for industrial battery research and development activities.

## The importance of cell area and format in battery performance reporting

Every cell format is important in battery research and development. However, the data obtained from each format must be evaluated within the boundaries defined by its design and the size of the tested electrodes. Uncritically extrapolating the performance of prospective commercial large-area cells from data generated with smaller formats often overlooks issues that become apparent only during scaling up. When going to mass production, such assumptions can lead to a substantial waste of time, materials, energy and money—far exceeding the resources required to produce a small number of commercial-like cells upfront.

**Fig. 1:**
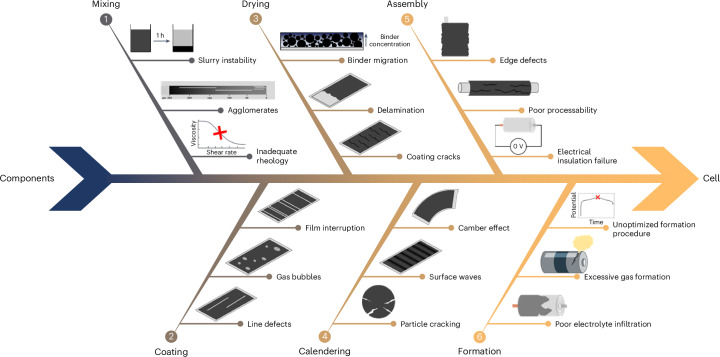
The various phases of electrode production and cell preparation. Fishbone diagram showing possible sources of defects and issues in the phases of electrode production (from 1 to 4), cell assembly (5) and formation (6).

In fact, upscaling new materials or processes developed at laboratary scale presents a series of challenges that are frequently neglected when the objective is limited to producing mAh-scale cells (that is, with TRL ≤3)^17,18^. As illustrated in Fig. 1, on the way from individual components (active materials, separator, electrolyte, current collector, casing and so on) to the finished cell, problems generally arise during slurry mixing and coating, electrode drying and calendering, and cell assembly and formation^19,20^. While not having the possibility here to discuss these challenges individually, we want to emphasize that the majority of the issues shown in Fig. 1 has a substantial impact on the battery performance and can be fully detected when assembling Ah-level large-area cells^20^. However, according to a recent peer-reviewed publication that analysed more than 13,000 scientific publications on batteries, only about 28% of the research articles reporting the electrode composition specify the area of the electrodes^21^. A related study on the same dataset suggests that even fewer accurately describe the cell format and design (for example, coin cell, pouch cell and so on)^22^.

We routinely use coin cells (approximately 2–6 mAh) to evaluate the specific capacity, initial coulombic efficiency and discharge rate capability, as they are simple and quick to assemble and test. Typically, for an initial assessment of the Li-ion storage capability, coin cells are assembled using the investigated active materials at the working (positive) electrode and lithium metal at the counter (negative) electrode, along with the same electrolyte solution that is used in upscaling experiments with larger formats. However, the reliability of cycle life data obtained from coin cells is questionable, as it has been shown to depend on highly variable parameters, such as the type of casing steel and the applied pressure, that is, the stack height^23,24^, which are independent of the materials studied. In addition, the misalignment of positive and negative electrodes can easily occur^25^, and the high perimeter-to-area ratio of small-area electrodes indicates a strong, detrimental influence of the electrodes’ edges on cell performance^26^. While these issues may still allow researchers to compare the results from coin cells assembled in similar conditions within their laboratories, comparisons of results obtained in different laboratories, where the assembling quality is not known, should be approached with caution. Although coin cells are usually the preferred format for low-TRL studies to characterize new battery materials, they should be considered inadequate predictors of cycling stability in scaling-up studies involving TRL ≥4 cells. Moreover, high-current-rate experiments should also be critically evaluated due to the large resistance of the coin cell set-up and, in the case of Li metal coin cell tests, the overpotential caused by the lithium metal counter/negative electrode, especially when testing working/positive electrodes with high areal capacity^23,24^.

Single-layer or double-layer pouch cells (approximately 50–200 mAh) are the next-size battery test vehicle in our workflow^27^. They can be conveniently assembled using a relatively small amount of active material and even hand-coated electrodes. Their larger area allows a more reliable assessment of long-term cycling stability^28^. These data can be used to infer the behaviour of larger cells, but with some important limitations to consider, such as the still relatively high perimeter-to-area ratio, the impact of the negative electrode overhang size on the total cell area, and a reduced temperature increase during cycling compared with Ah-scale cells^12,28^. In addition, pouch cells lack standardization in terms of dimensions and area, as they do not have a standardized hard case to constrain their size, unlike cylindrical and prismatic cells. Importantly, multilayer stacked pouch cells, that is, pouch cells whose electrodes are cut in multiple pieces with same dimensions and piled up^29^, do not ensure that the electrodes used during the assembly are consistently of high quality, as only the best sections of the electrode may be selected and cut for the cell assembly. This issue is reduced in large wound cells, as electrodes with lengths over 1 m are used in Ah-scale cells, which must meet strict production quality standards to ensure reliable cycling data. To ensure the reproducibility of battery testing, it is necessary to assemble a relevant amount of such wound cells where the double-sided electrodes must be homogeneously coated over a length in the order of 100 m to 1,000 m (refs. ^12,29^). Furthermore, winding induces stress on the electrodes due to the curvature around the cell’s winding mandrel, requiring them to have more stringent mechanical properties (for example, adhesion and cohesion) compared with stacked pouch cells^12,30^. Wound cell formats typically have predefined casings, such as 18650, 21700, 4680 and so on, for cylindrical cells, or VDA (acronym for *Verband der Automobilindustrie*, the German Association of the Automotive Industry) PHEV1, VDA PHEV2 and so on, for prismatic cells^29^. Standardized dimensions facilitate the comparison of energy density and performance across different cells from various research institutions or manufacturers. Nevertheless, they require more advanced equipment for their assembly and cycling, such as winding machines and high-current testing equipment. It is important to notice that, for applied testing currents >1 A, the thermal behaviour and the resistance of current collectors and cell’s connectors start acting as stumbling blocks for the delivery of adequate cell performance, unlike in smaller cell formats, where such high currents are usually not applied.

**Fig. 2:**
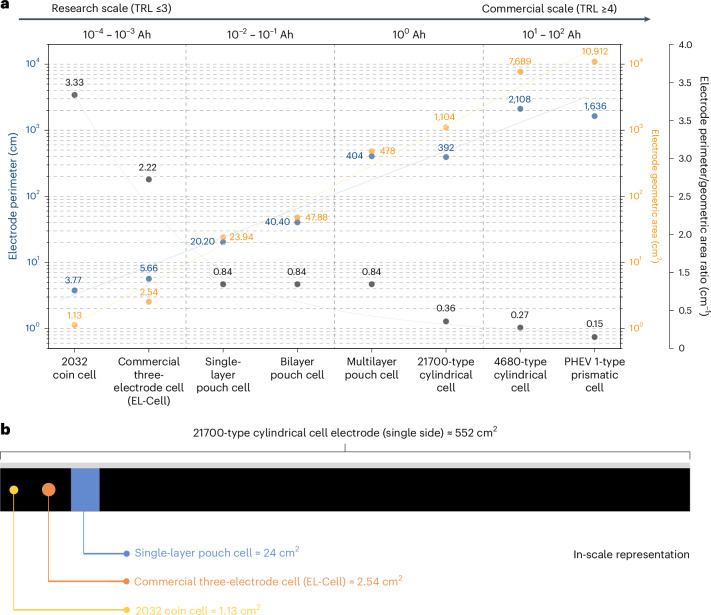
Variation of electrode geometric areas and perimeters moving from low-TRL to high-TRL battery research. **a**, The electrode perimeter (blue), electrode geometric area (yellow) and perimeter/geometric area ratio (grey) for different cell formats. Information regarding the assumed dimensions, number and type of electrodes for each cell format is provided in Supplementary Note 1. The dashed lines serve as a guide for the eyes. **b**, A schematic illustration with an in-scale comparison of the size of a typical 21700-type cylindrical cell electrode, a single-layer pouch cell electrode and disk-shaped electrodes for commercially available three-electrode cells (EL-Cell) and 2032 coin cells. For the 21700-type cylindrical cell electrode, the area of only one of the two coated sides is reported, for a better comparison with the other single-side coated electrodes.

Figure 2a shows the geometrical area and perimeter of electrodes for various types of laboratory-scale and industrial-scale cell configurations we assemble and test. Moving from the left to the right of the graph, it can be noticed how the values of electrode perimeter and geometrical area change when transitioning from basic low-TRL research to high-TRL commercial scale (data and methods used to calculate the geometrical area and perimeter values for each type of cell can be found in Supplementary Note 1). The geometrical area and perimeter span four to five orders of magnitude when transitioning from a 2032 coin cell to a prismatic cell. Whereas only a few square centimetres of electrode are needed for a coin cell, commercial cell formats require large, high-quality, defect-free electrodes to attain the intended battery performance and ensure data reliability. Based on the assumptions used for the calculations for Fig. 2a, 21700-cell electrodes are approximately 1 m long, while electrodes for a 4680-cell measure more than 5 m in length. This means that, for building these large-electrode-area cells, the absence of the issues illustrated in Fig. 1 must be ensured over multiples of these electrode lengths even at pilot scale, not to mention mass manufacturing. In general, regardless of the cell format used, ensuring the coating of high-quality, uniform electrodes should be a primary focus in battery research studies, and it is of paramount importance when aiming to produce and test cells with TRL ≥4.

To better visualize this size difference, Fig. 2b shows a scaled comparison of a typical electrode for a 21700 cylindrical cell versus electrodes for single-layer pouch cells, commercially available three-electrode cells (EL-Cell) and 2032 coin cells. From this schematic, it is evident that the geometrical area of the electrodes in coin cells is very limited compared with those used in cylindrical cells. The same consideration can be applied to electrodes used in single-layer pouch cells. Even in the case of multilayer pouch cells assembled in a stacked configuration, a selection of the most homogeneous negative and positive electrodes is still possible, as the individual electrodes in small-area pouch cells are usually much smaller than those in cylindrical or prismatic cells, where electrodes are wound rather than stacked^30^. This electrode selection for pouch cell assembly increases the scrap rate and hinders the identification of issues during the electrode production phase. Therefore, large-area, non-stacked cells can more reliably ensure that the used electrodes are free of defects that could negatively affect a cell’s electrochemical energy storage performance.

In Fig. 2a, we also show the electrode perimeter-to-geometric area ratio for each cell format, which can be used to assess the influence of electrode edges on cell behaviour. The edge region plays a critical role in the cell’s stability as it is a hotspot for lithium deposition at the negative electrode during battery operation^31^. Moreover, cutting electrodes into the desired shape can produce burrs and dust at the edges, increasing the risk of piercing the separator and causing local short circuits^30^. Therefore, a lower perimeter-to-geometric area ratio indicates better electrode quality and fewer defects. While a 12-mm-diameter coin cell electrode has a perimeter-to-area ratio of 3.33 cm^−1^, the same ratio for a double-side coated 6 × 92 cm^2^ 21700 cylindrical cell electrode is 0.36 cm^−1^, about ten times lower. The choice of large-area cells becomes then even more important, as it minimizes the adverse effects of edges on battery performance. With the detrimental effect of the electrodes’ edges, the actual performance of the cell will not align with the intended performance, because a higher number of edges increases the likelihood of defects and, hence, the possibility of triggering of failure mechanisms. We would also like to emphasize that, in the case of multilayer stacked pouch cells, increasing the number of layers (and, therefore, the capacity and area) does not result in a decrease in the perimeter-to-geometric area ratio, as the selected electrodes maintain the same geometric characteristics.

## Analysis of the battery performance of 21700 cylindrical cells prepared in the pilot plant

**Fig. 3:**
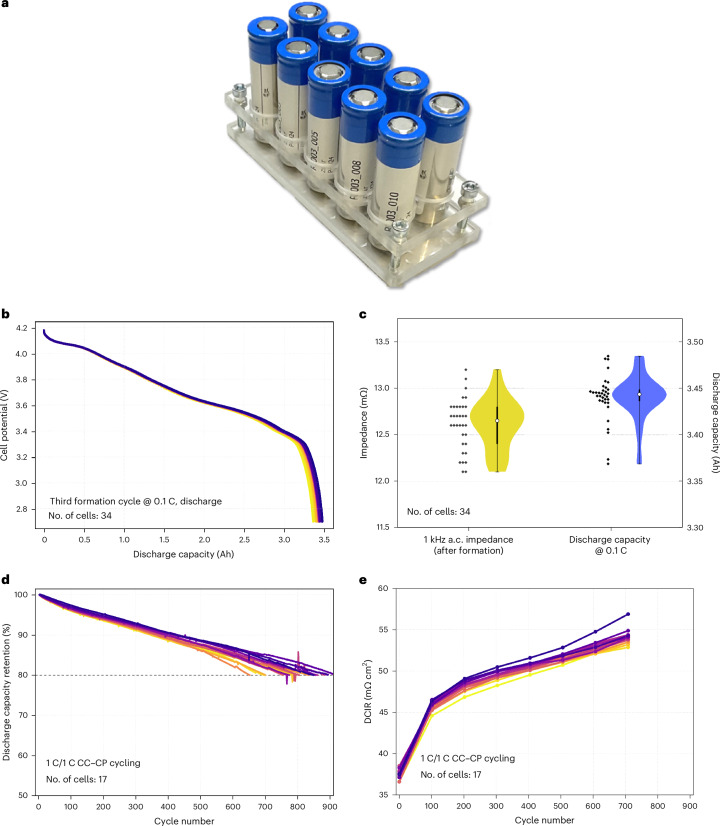
Testing of the pilot-scale-produced 21700 Li-ion cells. **a**, A photograph of some of the assembled and tested 21700 cylindrical Li-ion cells. **b**, A plot of cell potential versus discharge capacity of the third formation cycle at 0.1 C and 25 °C for 34 cylindrical cells, with each line, coloured in a scale from purple to yellow, representing a different cell. **c**, A violin plot showing the 1 kHz impedance in yellow (post-formation, 26 ± 1 °C) and discharge capacity in blue (third formation cycle at 0.1 C and 25 °C) for 34 cylindrical cells. The white dots indicate medians, black bars the interquartile range and whiskers the full range; narrower areas represent lower dispersion. **d**, Discharge capacity retention versus cycle number during 1 C/1 C CC–CP cycling at 25 °C for 17 cells, normalized to the first 1 C discharge, with each line, coloured in a scale from purple to yellow, representing a different cell. The 80% end-of-life capacity is marked with a dashed line. **e**, DCIR versus cycle number during 1 C/1 C CC–CP cycling at 25 °C for 17 cells, with each line, coloured in a scale from purple to yellow, representing a different cell. Data are shown up to cycle 708, the last with resistance measurements for all cells.

To demonstrate the level of consistency achievable at pilot scale with large-area wound cell formats, we present the results of an extensive experimental campaign, where 34 cylindrical 21700 cells (21 mm in diameter and 70 mm in height) were assembled and tested using the same active materials, electrode dimensions and cell design (Fig. 3a). Details regarding the materials, electrode production and cell assembly are provided in Methods.

Concerning the geometrical parameters of the cells, the positive electrode dimensions were 6 cm in width and 99.5 cm in length, double-side coated, with a total coated area of 1,194 cm^2^, while the negative electrode ones were 6.2 cm in width and 104.5 cm in length (with an overhang of 1 mm), double-side coated, resulting in a total coated area of 1,296 cm^2^. After laser ablation and tab welding^31^, the overall geometric active area of the cell was 1,166 cm^2^. In Table 1, we report these data with other relevant cell parameters.

**Table 1:** Relevant electrode and cell parameters for the 21700-type cylindrical cells reported in the present analysis

Parameter	Negative electrode	Positive electrode
Electrode length	104.5 cm	99.5 cm
Electrode width	6.2 cm	6.0 cm
Electrode coated area	1,296 cm^2^	1,194 cm^2^
Electrode areal capacity^a, b^	3.60 mAh cm^−2^	3.12 mAh cm^−2^
Electrode coating thickness^a^	71 µm	48 µm
Electrode coating density^a^	1.56 g cm^−3^	3.33 g cm^−3^
Electrode coating porosity^a^	27.5%	24.4%
No. of electrodes per cell	1 (double-side coated)	1 (double-side coated)

Parameter	Cell
Cell type	21700-type cylindrical cell
Cell geometric active area	1,166 cm^2^
Cell mass^c^	63.75 ± 0.33 g
1 kHz cell impedance	12.6 ± 0.3 mΩ
Cell discharge capacity @ 0.1 C	3.44 ± 0.03 Ah
Cell energy @ 0.2 C	11.81 ± 0.09 Wh
Cell specific energy @ 0.2 C^d^	185.27 ± 1.76 Wh kg^−1^
Cell energy density @ 0.2 C^e^	487.18 ± 3.87 Wh l^−1^

^a^Referred to one of the two coated sides. ^b^Areal capacity obtained by multiplying the electrode active material mass loading by the specific capacity of the electrode active material, measured in Li metal coin cells at 0.1 C and 26 ± 1 °C (208 mAh g^−1^ between 3 V and 4.3 V for the positive electrode material, 340 mAh g^−1^ between 0.01 V and 1.5 V for the negative electrode material). ^c^Mass of the finished cell before formation, including electrodes and electrolyte. ^d^Value calculated considering the cell mass reported in this same table. ^e^Value calculated considering the volume of a 21700 cylindrical cell (21 mm diameter by 70 mm height).

Figure 3b shows the cell potential–capacity curves during the discharge of all 34 cells in the third formation cycle @ 0.1 C (the same chart is reported in terms of specific capacity in Supplementary Fig. 1). On average, the discharge capacity during this cycle was 3.44 ± 0.03 Ah. This capacity served as the reference value for the 1 C current, which was set at 3.44 A. The 1 kHz impedance of the cell after formation, measured to assess the electric resistance, yields a value of 12.6 ± 0.3 mΩ. The numerical distributions of these two parameters are shown in Fig. 3c as a violin plot. A similar plot is found in Supplementary Fig. 2, showing the distributions of cell mass (63.75 ± 0.33 g) and discharged energy at 0.2 C (11.81 ± 0.09 Wh), resulting in an average cell specific energy of 185.27 ± 1.76 Wh kg^−1^ and an energy density of 487.18 ± 3.87 Wh l^−1^ at 0.2 C current rate. All these parameters indicate a high level of homogeneity in the electrodes used in terms of areal mass loading and capacity, as well as uniform impedance among the cells. This confirms the satisfactory level of reproducibility of the pilot-scale cylindrical cell manufacturing capabilities used.

Out of these 34 cells, 17 underwent further testing in a 1 C/1 C long-term cycling stability experiment at 25 °C, with a 0.1 C check-up cycle every 100 cycles. Check-up cycles at low current are implemented to verify the residual capacity of the cell without influence from the overpotentials and the temperature increase caused by the relatively high cycling currents. During these check-ups, the direct current internal resistance (DCIR) was also measured at 100% state of charge with a 30-s current pulse. Besides demonstrating a cell’s power capability^32^, the DCIR metric can also reveal variability among cells. In high-quality cells, the evolution of their DCIR during cycling should be uniform and not excessive. In fact, it is also a valuable indicator for monitoring cell health and can serve as an additional criterion for determining end of life^33^. The results of this testing procedure are shown in Fig. 3d,e and Supplementary Fig. 3. More variability among the cell performances can be observed in terms of cycling stability compared with the formation cycles, with a few cells showing a greater capacity fading than the average. In fact, the number of cycles at which this group of cells reaches the end-of-life criterion (that is, 80% of the beginning-of-life 1 C capacity) is 800 ± 73, but three of them reach this criterion between 650 and 700 cycles (see Supplementary Fig. 4, where the distribution of this quantity is visualized). If we do not consider these three outlier cells in the statistics, the 80% of capacity retention is reached after 825 ± 51 cycles. Regarding the DCIR, the average resistance at the first cycle is 37.5 ± 0.5 mΩ cm^2^ and increases to 54.1 ± 0.8 mΩ cm^2^ after about 700 cycles. These results align with the state-of-the-art reports on ageing studies of cylindrical commercial Li-ion cells, which utilize similar materials to those we used^34,35^. It should be pointed out that, in our case, some outlier cells are expected due to variabilities in the non-fully automated cells’ manufacturing pilot plant we used.

## Conclusions

In this Analysis, we discussed how large-area wound cells, such as 21700 cylindrical cells, provide greater reliability regarding the obtained electrochemical data compared with other small-area formats. This improvement is mainly associated with the ability of the large-area electrodes to minimize issues related to edge effects and to accurately reflect the quality of the manufactured electrodes. We hope that the results presented in this analysis will serve as a benchmark and demonstrate that consistent data can be obtained for Li-ion cells with TRL ≥4, prepared in a pilot plant for electrode manufacturing and cell assembly. We advocate that studies at this scale should include detailed information similar to the data presented in Table 1 and report the distribution of relevant cell parameters.

At the same time, it is important to stress that data obtained from Li-ion cells with TRL ≥4 should not be interpreted as a dismissal of other cell formats with lower TRL. Coin and small-area pouch cells (whether single- or multilayer) play a crucial role in every academic or industrial battery research laboratory. However, the data obtained in cells using low-area electrodes must be interpreted within the appropriate context to prevent excessive extrapolation^8^. Scaling up processes involves challenging engineering and technology activities, representing a fundamental aspect of scientific discovery. The ultimate impact of newly proposed concepts and ideas can only be fully understood and appreciated when applied in a practical environment; this process often drives innovation in ways that are not initially anticipated.

We advocate for stronger collaboration between academia and industrial research institutes to upscale and validate results obtained in laboratory-scale small-area Li-ion cells (that is, at TRL ≤3) to more practical and industrially relevant cell formats. Doing so may allow the gap between academic and industrial research to be bridged in the future, promoting coordinated progress in battery research and development.

## Methods

### Electrode preparation and cell assembly

The electrode slurries were mixed in air conditions in a 10-litre vessel using a planetary mixer equipped with butterfly and crossbeam stirrers (Netzsch). The electrode slurry was applied through a comma bar coating system on a pilot coating line (LACOM GmbH) with four separate drying zones along its 8-m length. For the preparation of the positive electrode slurry, *N*-methyl-2-pyrrolidone (Oqema, >99.5%) was used as the solvent. For the preparation of the negative electrode slurry, deionized water (<2.0 µS cm^−1^) was used as solvent. The positive electrode slurry was coated onto 15-µm-thick aluminium foil (Hydro, AA1100), and the negative electrode slurry was coated onto 10-µm-thick copper foil (JX Nippon, HS1200, Cu-0.12%Sn). Both positive and negative pilot electrodes were coated to a width of 80 mm. Drying of the electrodes was performed at temperatures ranging from 50 °C to 110 °C in the convection oven of the coating line at ambient pressure. To determine the electrode porosity *ε*, the density of the electrode without porosity *ρ*_el_ was first calculated using the mass fractions *x*_*i*_ and densities *ρ*_*i*_ of its components

$${\rho }_{{{\mathrm{el}}}}=\frac{1}{\sum \frac{{x}_{i}}{{\rho }_{i}}}.$$

Next, the density measured after calendering *ρ*_el,cal_ was divided by electrode density without porosity. The electrode porosity was then calculated as one minus this ratio

$$\varepsilon =1-\frac{{\rho }_{{{\mathrm{el}}},{{\mathrm{cal}}}}}{{\rho }_{{{\mathrm{el}}}}}.$$

The positive electrode active material was LiNi_0.83_Co_0.12_Mn_0.05_O_2_ (NMC 811, Targray), with an average particle size of *D*_50_ = 11.8 µm (*D*_10_ = 5.6 µm, *D*_90_ = 21.2 µm) and a surface area (calculated via Brunauer–Emmett–Teller theory) of 0.4 m^2^ g^−1^ (values provided by the supplier). The positive electrode composition consisted of 93% active material, 3% carbon black (CB, Super C65, Imerys, >99.8%, *D*_50_ < 50 nm), 1% graphite (SFG6L, Imerys, >99.8%, *D*_50_ = 3.5 µm) and 3% polyvinylidene fluoride (Solef 5130/1001, Solvay Solexis, >99.999%). The dry slurry-coated positive electrode exhibited an areal capacity of approximately 3.12 mAh cm^−2^ (active material loading ~14.89 mg cm^−2^), and it was calendered (Mathis AG) with 60 ton, 68 µm of gap, at 1 m min^−1^ and 100 °C to achieve an average density of 3.33 g cm^−3^ and an average single-side thickness of 48 µm, excluding the Al current collector (calculated porosity 24.4%). For the porosity calculation, densities of 4.80 g cm^−3^ for the NMC, 1.85 g cm^−3^ for the CB, 2.26 g cm^−3^ for the graphite, and 1.78 g cm^−3^ for the polyvinylidene fluoride were assumed. Before cell assembly, the positive electrode was further dried after calendering at 110 °C for 16 h in a vacuum oven (PINK GmbH Thermosysteme).

The negative electrode formulation comprises 94% graphite (Showa Denko, *D*_50_ = 11.4 µm), 2% CB (Super C65, Imerys, >99.8%, *D*_50_ < 50 nm), 2% carboxymethyl cellulose (MAC 500LC, Sunrose, average molecular weight 500,000 g mol^−1^) and 2% styrene-butadiene rubber (Zeon, 40 wt.% aqueous dispersion) binders. This pilot-scale negative electrode exhibited an areal capacity of approximately 3.60 mAh cm^−2^ (active material loading ~10.46 mg cm^−2^), resulting in a negative-to-positive ratio for the Li-ion cells of about 1.15. The dry slurry-coated negative electrode was calendered (Mathis AG) with 60 ton, 108 µm of gap, at 1 m min^−1^ and 40 °C to an average density of 1.56 g cm^−3^ and an average single-side thickness of 71 µm, excluding the Cu current collector (calculated porosity 27.5%). For the porosity calculation, true densities of 2.26 g cm^−3^ for the graphite, 1.85 g cm^−3^ for the CB, 1.60 g cm^−3^ for the carboxymethyl cellulose and 0.94 g cm^−3^ for the styrene-butadiene rubber were assumed. The negative electrode was further dried after calendering at 130 °C for 16 h in a vacuum oven (PINK GmbH Thermosysteme) before cell assembly.

State-of-the-art equipment and a dry room (80 m^2^, dew point <−60 °C) were used for the assembly and testing of 21700-type cylindrical lithium-ion batteries. The equipment included tools for electrode slitting, laser ablation, ultrasonic welding of tabs, automated electrode winding, cell grooving, electrolyte filling and cell crimping^13,14^.

After tab welding, the jelly rolls—that is, the wound assemblies of the positive electrode, separators and negative electrode—were produced using a semi-automatic winding machine (Sovema). A customized Celgard microporous membrane was used as separator (thickness 16 µm, width 65 mm). Before inserting the jelly roll into the 21700 stainless-steel can, a high-potential test (Flüke 1587) was performed to check the isolation of the separator within the jelly roll by applying a 100 V d.c. potential between the negative and positive tabs for 3 s and reading the measured resistance, which should be higher than 50 MΩ. Subsequently, the positive and negative electrode tabs were welded to the bottom of the can and the lid via electric resistance welding and ultrasonic welding, respectively. The cylindrical cells were filled with 7 ml (approximately 2 ml per Ah) of 1.4 M lithium hexafluorophosphate (LiPF_6_) in ethylene carbonate:dimethyl carbonate (3:7 wt.%) + 2 wt.% vinylene carbonate electrolyte solution (Gotion, H_2_O 4.4 ppm, HF 21.5 ppm) inside an Ar-filled glovebox (O_2_ <0.1 ppm, H_2_O <0.1 ppm) and then crimped in the same environment.

### Electrochemical measurements

All electrochemical measurements were carried out with BaSyTec CTS cyclers in a climate chamber (Vötsch LabEvent) set at 25 °C, with additional monitoring of individual cell temperatures with one K-type thermocouple per cell placed in the middle of its lateral surface and connected to the cycler. The formation procedure involved charging at 0.1 C (constant current–constant potential (CC–CP) protocol with charge cut-off at 0.05 C) and discharging (applying a CC) for three cycles between 4.2 V and 2.5 V of cell potential, estimating the 1 C current with the cell capacity calculated considering only the positive electrode contribution. After formation, the cell impedance was measured at 1 kHz at open circuit potential (using an HIOKI 3554 battery internal resistance tester), followed by cell rating to determine the specific energy and the energy density. The cell rating was performed with the same protocol of the formation procedure but with a 0.2 C current, using the discharge capacity of the last formation cycle to obtain the actual 1 C current of each cell. During long-term cycling (1 C/1 C, charge CC–CP, charge cut-off at 0.05 C, discharge CC), every 100th cycle was designated as a check-up cycle carried out at 0.1 C charge/discharge rate. After the check-up cycle charging step, the cell was rested for 30 min to determine the open circuit potential in the fully charged state. The DCIR was then measured by applying a 1 A discharge pulse for 10 s, followed by 10 A discharge pulse for 1 s. To obtain the DCIR, the difference between the final recorded potential points of the 1 A and 10 A pulses (*E*_1_ and *E*_2_, respectively) is divided by the difference between the two currents (*I*_1_ and *I*_2_, respectively)

$${{\mathrm{DCIR}}}=\frac{{E}_{1}-{E}_{2}}{{I}_{2}-{I}_{1}}.$$

After that, the 0.2 C discharge step of the check-up cycle was resumed. These check-up cycles are omitted in Fig. 3d and Supplementary Fig. 3 for clarity, but they are reported in Supplementary Fig. 5.

## Online content

Any methods, additional references, Nature Portfolio reporting summaries, source data, extended data, supplementary information, acknowledgements, peer review information; details of author contributions and competing interests; and statements of data and code availability are available at 10.1038/s41565-025-01906-3.

## Supplementary information

Supplementary Information Supplementary Note 1 and Figs. 1–5.

## Data Availability

Data will be made available on request.
