# Supplementary material for: From small batteries to big claims
Source: Nat Nanotechnol. 2025 Apr 11;20(7):970–6. doi: 10.1038/s41565-025-01906-3 (PMC12267060; doi:10.1038/s41565-025-01906-3)
Supplement: Supplementary file 1 — Supplementary Note 1 and Figs. 1–5. [file 41565_2025_1906_MOESM1_ESM.pdf]

nature nanotechnology

Supplementary information https://doi.org/10.1038/s41565-025-01906-3

From small batteries to big claims

In the format provided by the authors and unedited

Table of Contents

Supplementary Note 1: Technical considerations for the preparation of Figure 2a 2
Supplementary Figures.. 3
References 8

Supplementary Note 1: Technical considerations for the preparation of Figure 2a

To obtain the values of cell geometric area and perimeter reported in **Figure 2a** in the main text, we made the following considerations:

- **Coin cell**: we assumed a 12 mm diameter for the coin cell electrode, a standard that is conventionally used in low-TRL research. The electrodes are single-side coated.
- **Three-electrode cell**: we selected a diameter of 18 mm for the three-electrode cell, as it is the standard diameter of the commercial PAT-Core cell from the company EL-CELL®. The electrodes are single-side coated.
- **Single-layer pouch cell**: positive electrode width = 6.3 cm; positive electrode length = 3.8 cm. We used these values as they are the standard dimensions of a pouch cell cathode we use in our laboratories. The electrodes are single-side coated.
- **Bi-layer pouch cell**: we doubled the values used for the single-layer pouch cell. The electrodes at the edge of the pouch cell stack are single-side coated, while the one in the middle is double-side coated.
- **Multi-layer pouch cell**: we multiplied by a factor of 20 the values used for the single-layer pouch cell. The electrodes at the edge of the pouch cell stack are single-side coated, while the ones in the middle are double-side coated.
- **21700-type cylindrical cell**: positive electrode width = 6 cm; positive electrode length = 92 cm. We took the dimensions of the 21700 cell assembled with the electrodes, current collectors and separator as reported in the literature.[1] The electrodes are double-side coated.
- **4680-type cylindrical cell**: positive electrode width = 7.4 cm, positive electrode length = 520 cm. We simulated the design of a tabless 4680 cell with the software Simcenter Battery Design Studio,[2] using the thickness of electrodes, current collectors and separator as reported in the literature.[1] The electrodes are double-side coated.
- **PHEV 1 prismatic cell**: positive electrode width = 13.8 cm, positive electrode length = 395 cm. We used the geometrical characteristics of the PHEV 1 cell assembled with the electrode, current collectors and separator as reported in the literature.[1] The electrodes are double-side coated.

Assuming the presence of an overhang of the negative electrode on the positive electrode, as in the case of commercial cells, the positive electrode size was considered to determine the dimensions in the calculation of the area and perimeter of these cell configurations.

Supplementary Figures

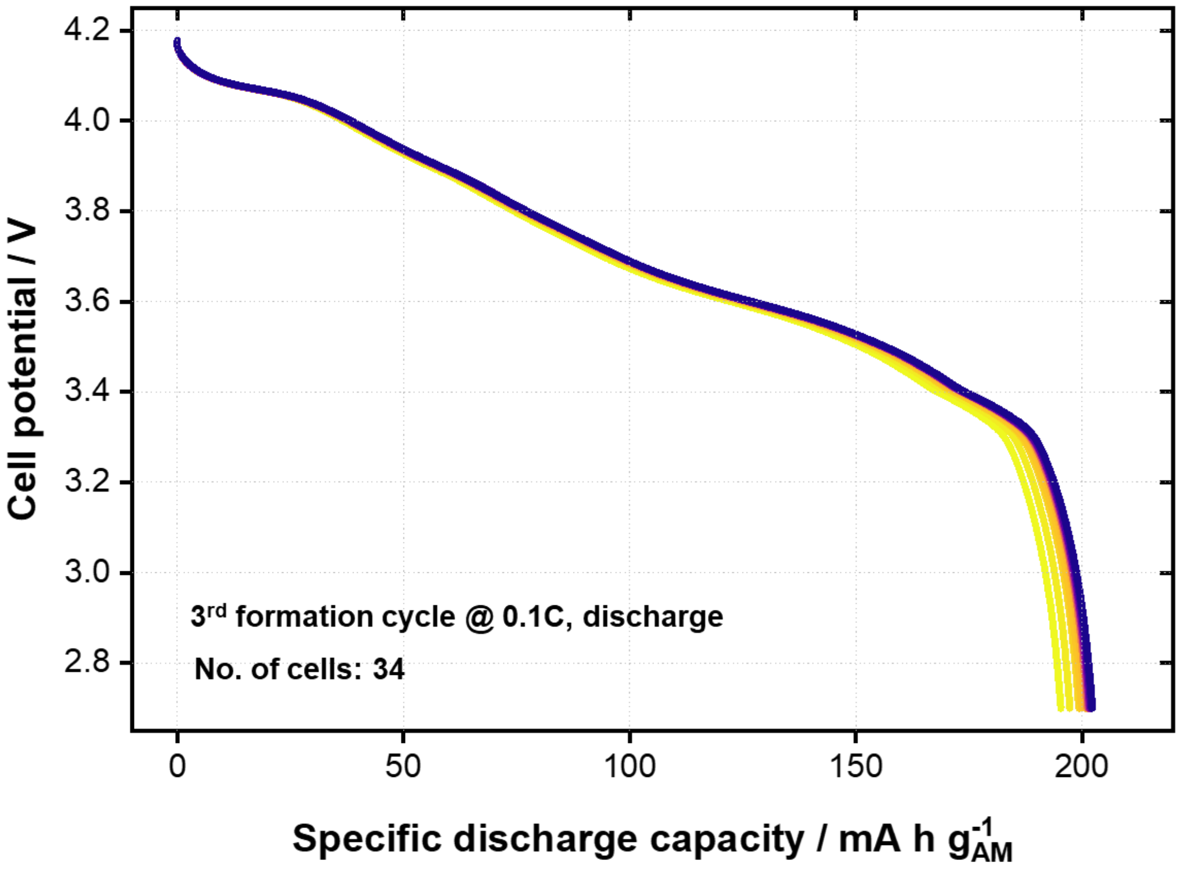

Supplementary Figure 1 – Cell potential vs. specific discharge capacity plot of the 3rd formation cycle @ 0.1C and 25°C of all the 34 cylindrical cells, with each line colored in a scale from purple to yellow, representing a different cell (see Figure 3b). The specific discharge capacity was calculated using the active material mass of the positive electrodes as reference mass.

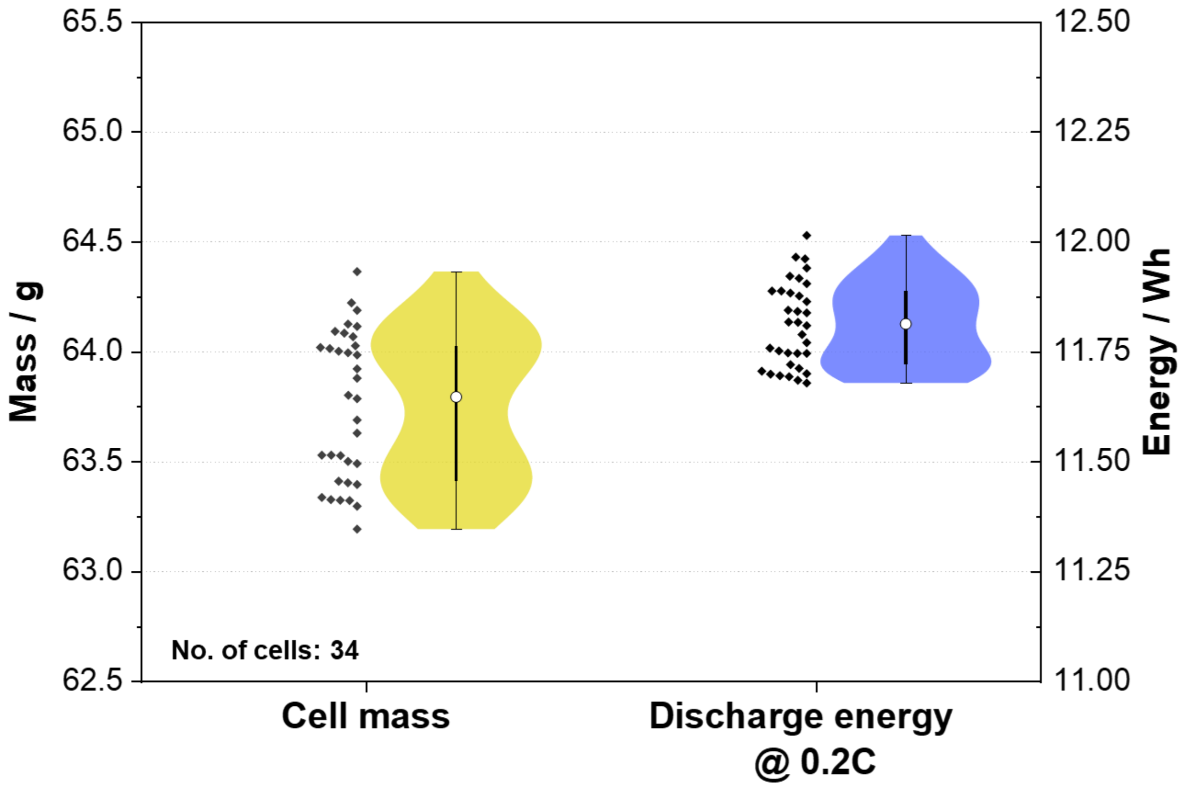

Supplementary Figure 2 – Violin plot of the distribution of the cell mass (in yellow) and of the discharge energy @ 0.2C (in blue) of all the 34 cylindrical cells. In the violin plot, the white dot represents the median value, the black bar is the 25- 75% interquartile range, and the whiskers are the minimum and maximum values of the distribution. The narrower is the area representing the quantity distribution, the smaller is the dispersion between the values. The single data points are also reported next to the distribution.

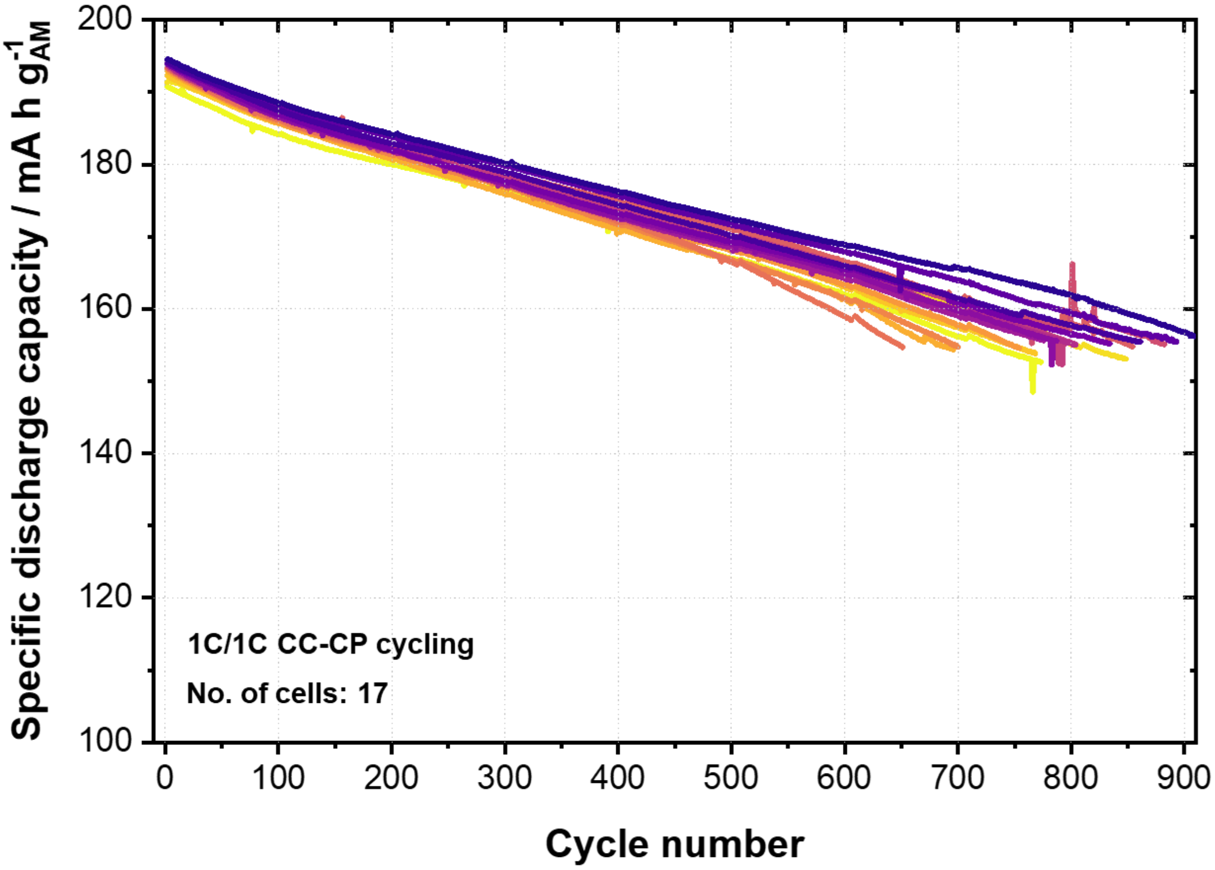

Supplementary Figure 3 – Specific discharge capacity vs. cycle number during the 1C/1C constant current-constant potential (CC-CP) protocol of 17 cylindrical cells tested for long-term cycling stability at 25°C, with each line colored in a scale from purple to yellow, representing a different cell. The specific discharge capacity was calculated using the active material mass of the positive electrodes as reference mass. The cells were stopped after reaching 80% of their initial discharge capacity at 1C (see Figure 3d).

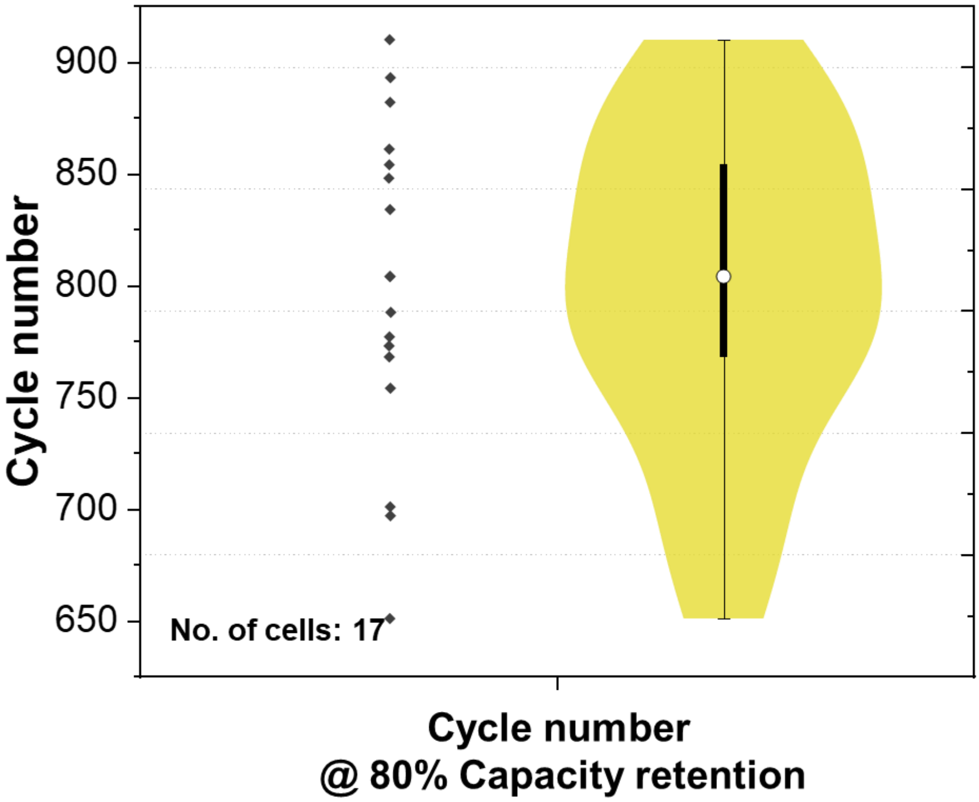

Supplementary Figure 4 – Violin plot of the distribution of the cycle number at which 80% discharge capacity retention is reached during long-term cycling (data from Figure 3b and Supplementary Figure 3). In the violin plot, the white dot represents the median value, the black bar is the 25-75% interquartile range, and the whiskers are the minimum and maximum values of the distribution. The narrower the area representing the quantity distribution, the smaller the dispersion between the values. The single data points are also reported next to the distribution.

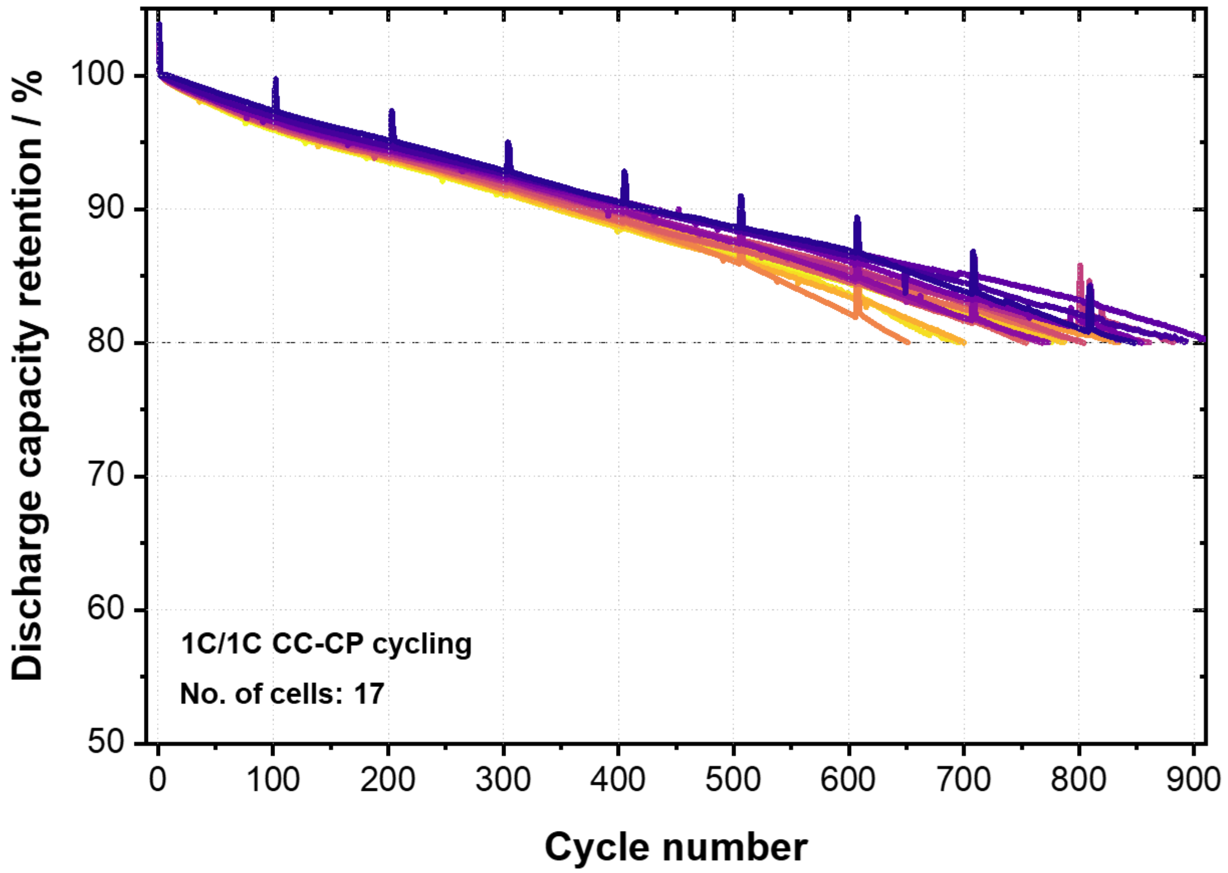

Supplementary Figure 5 – Discharge capacity retention vs. cycle number during 1C/1C CC-CP cycling at 25°C for 17 cells, normalized to the first 1C discharge, with each line colored in a scale from purple to yellow, representing a different cell. The 80% end-of-life capacity is marked with a dashed line. Unlike in Figure 3d, here the check-up cycles were not omitted.

References

1. Waldmann, T. *et al.* A direct comparison of pilot-scale Li-ion cells in the formats PHEV1, pouch, and 21700. *J. Electrochem. Soc.* **168**, 090519 (2021).

2. Simcenter Battery Design Studio. *Siemens Digital Industries Software*. https://plm.sw.siemens.com/en-US/simcenter/fluids-thermal-simulation/battery-design-studio/. Accessed on 17.10.2024.
